# Neutrophil activation may trigger tau burden contributing to cognitive progression of chronic sleep disturbance in elderly individuals not living with dementia

**DOI:** 10.1186/s12916-023-02910-x

**Published:** 2023-06-06

**Authors:** Lin Sun, Jie Zhang, Wei Li, Jianhua Sheng, Shifu Xiao, Alzheimer’s Disease Neuroimaging Initiative

**Affiliations:** 1 Department of Psychiatry, Alzheimer’s Disease and Related Disorders Center, Shanghai Mental Health Center, Shanghai Jiao Tong University School of Medicine, No. 600 South Wanping Road, Xuhui District, Shanghai, China; 2 Key Laboratory of Spine and Spinal Cord Injury Repair and Regeneration of Ministry of Education, Orthopedic Department of Tongji Hospital, School of Medicine, Tongji University, Shanghai, China

**Keywords:** Chronic sleep disturbance, Cognitive progression, Neutrophil, Tau burden, Alzheimer’s disease

## Abstract

**Background:**

To investigate the complex connection between chronic sleep disturbance (CSD) and cognitive progression.

**Methods:**

The Alzheimer’s Disease Neuroimaging Initiative (ADNI) database was used to assign 784 non-dementia elderly into two groups: a normal sleep group (528 participants) and a CSD group (256 participants) via the Neuropsychiatric Inventory (NPI)-sleep subitem. Blood transcriptomics, blood neutrophil, cerebrospinal fluid (CSF) biomarkers of Alzheimer’s disease (AD), and neutrophil-related inflammatory factors were measured. We also investigated gene set enrichment analysis (GSEA), Cox proportional hazards model for risk factors, and mediation and interaction effects between indicators. Cognitive progression is defined as the progression from cognitively normal to mild cognitive impairment (MCI)/dementia or from MCI to dementia.

**Results:**

CSD could significantly affect cognitive function. The activated neutrophil pathways for cognitive progression in CSD were identified by transcriptomics GSEA, which was reflected by increased blood neutrophil level and its correlation with cognitive progression in CSD. High tau burden mediated the influence of neutrophils on cognitive function and exacerbated the CSD-related risk of left hippocampal atrophy. Elevated neutrophil-related inflammatory factors were observed in the cognitive progression of CSD and were associated with brain tau burden.

**Conclusions:**

Activated neutrophil pathway triggering tau pathology may underline the mechanism of cognitive progression in CSD.

**Supplementary Information:**

The online version contains supplementary material available at 10.1186/s12916-023-02910-x.

## Background

More than 47 million people worldwide are affected by dementia, and the number is estimated to double every 20 years with the increases in life expectancy [1, 2]. Alzheimer’s disease (AD) accounts for 60 to 70% of all dementia cases [3], and the accumulation of toxic forms of β-amyloid (Aβ) and tau neurofibrillary tangles in the brain is considered the main pathological mechanism. According to epidemiological studies, sleep difficulties are significantly more common among the elderly, with a prevalence of 36 to 69% [4]. When left untreated, sleep difficulties are associated with severe adverse consequences, ranging from poor mental health to cardiovascular disease [5]. According to the Neuropsychiatric Inventory (NPI)-sleep subitem, chronic sleep disturbance (CSD) consists of 8 aspects, including difficulty falling asleep, superficial sleep, early awakening, interrupted sleep, abnormal nighttime behavior, excessive daytime sleepiness, getting up at night, and other nocturnal abnormal behaviors. A bidirectional relationship exists between sleep and neurodegenerative disorders, such as AD, caused by Aβ and tau pathologies [6]. Extended periods of wakefulness or sleep deprivation were found to regulate the extracellular release of both Aβ [7, 8] and tau [9, 10]. Besides Aβ pathology, another biologically potential mechanism linking CSD and dementia risk is inflammatory response activation, which is thought to be an early event associated with the onset and clinical course of AD [11]. The mechanism by which inflammation associated with AD pathology regulates the relevance of CSD to cognitive function remains unclear. A better understanding of the mechanism of the effect of CSD on cognitive progression can help identify potential targets for dementia prevention.

Previous studies have shown that sleep disturbance can influence the hypothalamus–pituitary–adrenal (HPA) axis and the sympathetic nervous system (SNS), which together alter the profile of proinflammatory gene expression [12], and induce increases in interleukin 6 (IL-6), C-reactive protein (CRP) [13], tumor necrosis factor α (TNF-α), interleukin 1β (IL-1β) [14], and vascular cell adhesion molecule-1 (VCAM-1) [15]. Growing evidences demonstrated that sleep disturbance links with systemic inflammation and that neutrophils, as a marker of the ongoing non-specific inflammation and the first-line innate immune cell, can induce the uncontrolled release of toxic substances including inflammatory cytokines and tissue-damaging materials [16]. The ratio of neutrophil-to-lymphocyte has been repeatedly shown to be associated with obstructive sleep apnea [16–18]. Activation of neutrophils induces the release of neutrophil extracellular traps (NETs), leading to vascular destabilization [19], breakdown of the blood–brain barrier (BBB) [20], and vulnerability of the brain to damage [21]. Meanwhile, inflammatory cytokines also promote vascular permeability, neutrophil adhesion, and migration [22]. These findings suggested the importance of neutrophils in CSD, and we would focus on the effects of neutrophils on the association of CSD and cognitive function. In the present study, the longitudinal analysis showed the association of CSD with cognitive progression, and transcriptomics enrichment analysis revealed the activated neutrophil pathways in cognitively progressive subjects with CSD, which was echoed by analyses on blood neutrophils. The correlation between the neutrophil pathway and brain tau burden may reveal the mechanism, by which neutrophil activation triggers tau pathology to impair cognitive function in CSD.

## Methods

### Subjects

The data on 2272 adults were retrieved from the Alzheimer’s Disease Neuroimaging Initiative (ADNI) database (http://adni.loni.usc.edu). ADNI is a multi-site dataset launched in 2003 and designed to test the clinical symptoms, imaging, genetic, and biochemical biomarkers of AD. Data collection and sharing in ADNI were approved by the institutional review boards of all participating institutions. Written informed consent was obtained from all participants or their guardians in accordance with the Declaration of Helsinki. The participants are older adults aged 55–90. Each participant had an in-person neuropsychological assessment interview at baseline and annual follow-up [23, 24]. The inclusion and exclusion criteria are shown in the flow chart (Additional file 1). The subjects in this study received an NPI-sleep assessment [25, 26] at least twice in follow-up visits, with an interval of 6 months. The participants reporting normal sleep at each follow-up were selected for the normal sleep group (*n* = 528), and the subjects reporting sleep difficulties at baseline and follow-up (≥ 2 times in total) were selected for the CSD group (*n* = 256). To avoid information bias, patients who reported sleep disturbance only once were excluded. Finally, a total of 784 non-dementia elderly were included in this study. Detailed NPI-sleep assessments are provided in Additional file 2.

Cognitive assessments included the Mini-Mental State Examination (MMSE), the Alzheimer’s Disease Assessment Scale-cognitive (ADAS-cog), the Functional Activities Questionnaire (FAQ), the ADNI-MEM for memory, and the Montreal Cognitive Assessment (MoCA). In the ADNI, cognitively normal subjects had MMSE scores of 24–30, a Clinical Dementia Rating (CDR) of 0, and no memory complaints. Subjects with mild cognitive impairment (MCI) had MMSE scores of 24–30, a CDR score of 0.5, an informant-reported memory complaint, and objective evidence of memory loss. Through follow-up, dementia was diagnosed with MMSE scores of 20–26, a CDR score of 0.5–1, and subjective memory concern as reported by the subject, study partner, or clinician. The detailed criterion can be found in Additional file 2.

Individuals had in-person interviews at baseline and follow-up per 6 months, and the follow-up time was up to 168 months (28 times). A total of 784 non-dementia elderly were involved in this study at baseline, which included normal cognitive and MCI subjects. Through follow-up, cognitively stable individuals were defined as (1) stable normal cognitive, (2) stable MCI, or (3) MCI reversing into cognitively normal. Meanwhile, cognitively progressive individuals were defined as (1) MCI progressing into dementia or (2) cognitively normal progressing into MCI or dementia.

### Routine blood test

After fasting overnight, the participants’ blood was collected in the morning using vacuum tubes with ethylene diamine tetraacetic acid (EDTA). The blood samples were then sent for analysis on the same day of collection for routine blood test, including blood neutrophil percentage.

### *APOE* genotype

DNA was extracted with the QIAamp®DNA Blood Mini Kit and amplified by the polymerase chain reaction (PCR) with forward primers 5′-ACGGCTGTCCAAGGAGCTG-3′ (rs429358) and 5′-CTCCGCGATGCCGATGAC-3′ (rs7412). *APOE* genotype was performed through restriction fragment length polymorphism (RFLP) technology.

### Hippocampal volume

Annual change rates of hippocampal volumes were measured longitudinally in 198 subjects who had ≥ 3 available MRI assessments provided by ADNI imaging data. The methodology details of measuring hippocampal volumes are in the Additional file 3 [27].

### CSF AD type and inflammatory biomarkers

Before analysis, all concentrations were normalized into *Z* scores, and outliers beyond ± 3*δ* were excluded. The CSF from 771 subjects had typical AD biomarkers, including Aβ_42_, p-tau, and t-tau proteins. These biomarkers were detected using a fully automated and highly standardized Roche Elecsys immunoassay. Meanwhile, the CSF from 189 subjects had inflammatory factors, including tumor necrosis factor receptor 1,2 (TNFR1,2); transforming growth factor 1,2,3 (TGFβ1,2,3); interleukin 6,7,21 (IL6,7,21); intercellular adhesion molecule 1 (ICAM1); and VCAM1. These inflammatory markers were detected using commercially available multiplex immunoassays (Millipore Sigma, Burlington, MA) modified for CSF analyte levels.

### Blood-based microarray profiling and analysis

The PAXgene Blood RNA Kit (Qiagen Inc., Valencia, CA, USA) was used to purify total RNA from the whole blood collected in a PAXgene Blood RNA Tube. The Affymetrix Human Genome U219 Array (Affymetrix, Santa Clara, CA, USA) was used for expression profiling in ADNI. The quality of gene expression data, including sample quality, hybridization, and overall signal quality, was analyzed using Affymetrix Expression Console software and Partek Genomic Suite 6.6. Raw expression values were pre-processed using the robust multichip average (RMA) normalization method.

Enrichment analysis was performed on 88 cognitively stable and 54 cognitively progressive subjects with CSD to investigate the activated pathways for cognitive progression. Gene set enrichment analysis (GSEA) [28] in R was used to screen the biological process (BP) of the Gene Ontology (GO) term through the c5.go.bp.v7.5.symbols.gmt in the Molecular Signatures Database (MSigDB) [28], the GO-Biological-Process-2018.txt in the Enrichr database as a reference gene set, and the Disease-Perturbations-from-GEO-up.txt in the Enrichr database [29] for the pathway term between the groups. A value of adjusted *p* < 0.05 was considered significant.

### Statistical analyses

The Mann–Whitney *U* test was used for continuous variables with non-normal distributions, while the chi-square test was used for categorical variables to test the differences between the groups.

The linear mixed-effects model depicted the effects of CSD on longitudinal clinical outcomes, including cognitive function and social activity function. The model included random slope and intercept terms for each participant. The effect of neutrophils on cognitive function was demonstrated using hierarchical regression. The risk factor of CSD for cognitive progression was predicted using a time-dependent Cox proportional hazards model. To control for confounding by multiple factors, such as tau burden including CSF t-tau and p-tau, and other psychiatric symptoms including depression, appetite, aberrance, irritability, disinhibition, elation, and agitation, were all included in the Cox regression model. To eliminate the difference caused by a range of cognitive function between normal and MCI, besides demographic factors, baseline global cognition score (MMSE) was incorporated in the Cox regression model to assess the cognitive progression risk of CSD or neutrophil. A restricted cubic spline (RCS) curve was used to explore the association between blood neutrophils and cognitive outcome. RCS allows threshold identification of neutrophils on cognitive progression risk. According to the threshold of 61.33, we then divided the cohort into two subgroups (low neutrophil group < 61.633% versus high neutrophil group > 61.633%). Additionally, a Kaplan–Meier curve was used to plot the risk of cognitive progression. To determine annual change rates in cognition and hippocampal volume, we used the fitted linear mixed models with MMSE, ADAS-cog, FAQ, MoCA, ADNI-MEM, and hippocampal volumes as dependent variables and time (years from baseline) as independent variables, controlling for random intercept and slope. Then, a slope for the annual change rate was created for each subject. Longitudinal analyses were restricted to subjects with at least 3 time points.

Routine blood test of neutrophil and CSF inflammatory factors including TNFR1,2; TGFβ1,2,3; IL-6,7,21; ICAM1; and VCAM1 were compared between the normal and CSD groups using the Mann–Whitney *U* test. The Benjamini–Hochberg correction was used to adjust the *p*-value.

We used analysis of covariance (ANCOVA) to test whether CSF t-tau or p-tau positivity aggravated the association between CSD and hippocampal atrophy, after controlling for the main effects of sleep, tau burden (CSF p-tau or t-tau), age, gender, education, *APOE*, and intracranial volume. p-tau positivity was defined as p-tau > 21.8 pg/mL, and t-tau positivity was defined as t-tau > 245 pg/ml. The *p*-value was corrected using a Bonferroni correction with an *α*-threshold of 0.025.

Mediation analysis was used to explore what pathways mediated the link between blood neutrophils and cognitive function. Through exploratory analysis, we assigned *X* as the blood neutrophil percentage, *M* (mediator) as brain tau burden (CSF t-tau or p-tau at baseline), and *Y* as the outcome (cognitive function and social activity function at 2-year follow-up). We then interpreted the total effect as the amplitude of neutrophils in cognitive progression, both directly and through tau pathology intermediates. Next, we estimated the effects of the tau burden on the cognitive outcome after observing the association for a potential mediator. Age, education, gender, and *APOE* genotype were included as covariates, and the total, direct, and indirect effects were estimated using model 4 (mediation) of the PROCESS macro by the bruceR package in R with a bootstrapping module of 1000 iterations.

The statistical significance of all tests had a *p*-value of < 0.05. All analyses were performed using SPSS 17.0 or R version 4.2.0.

## Results

### Participant characteristics

**Fig. 1:**
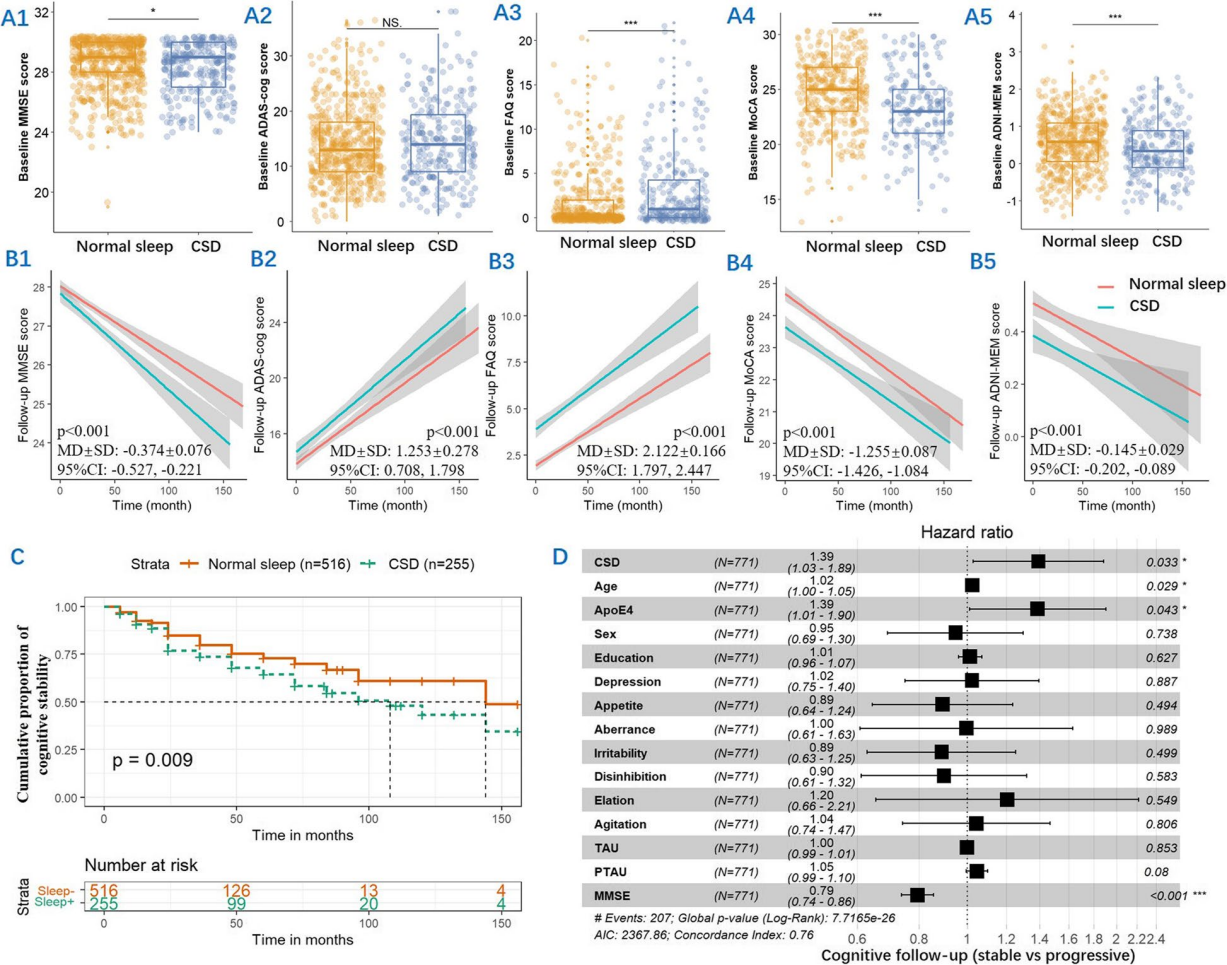
CSD may significantly increase the risk of cognitive decline. The significant differences in MMSE, FAQ, MoCA, and ADNI-MEM between the normal sleep and CSD groups at baseline, except for the ADAS-cog score. NS *p* > 0.05, *p < 0.05, ***p < 0.001 (**A1**–**A5**). The differences in MMSE, ADAS-cog, FAQ, MoCA, and ADNI-MEM at follow-up between the two groups based on the linear mixed-effects model after adjustment for age, education years, sex, and *APOE* status. There is no significant difference in the slope of the lines between the two groups (**B1**–**B5**). A Kaplan–Meier curve showed that the CSD group was associated with a higher risk of cognitive progression than the normal sleep group (**C**). The Cox proportional hazards model estimated the risk of cognitive progression in CSD after adjustment for age, education years, sex, *APOE* status, baseline MMSE, CSF t-tau, CSF p-tau, and multiple psychiatric symptoms (**D**)

A total of 784 elderly were included in the present study, and their characteristics from ADNI are summarized in Table 1. The participants were 72.91 ± 7.04 years old, had MMSE scores of 28.31 ± 1.72, and had a moderate education of 16.29 ± 2.64 years. Male participants accounted for 55.7% of the total participants. Subjects with CSD had less education, more impaired cognitive function, and lower social activity function (*p* < 0.05) (Table 1, Fig. 1A1–A5). Cognitively progressive elderly with CSD tended to be *APOE* ε4 carriers.

**Table 1:** Characteristics of participants

Characteristics	Normal sleep 1 (*n* = 528)		CSD 2 (*n* = 256)		*p* or adjusted *p* (*U* or *χ*^2^) (*η*^2^), 1 vs 2	*p* or adjusted *p* (*U* or *χ*^2^) (*η*^2^), 3 vs 4	*p* or adjusted *p* (*U* or *χ*^2^) (*η*^2^), 5 vs 6
	Cognitively normal 3	Cognitively progressive 4	Cognitively normal 5	Cognitively progressive 6			
**Baseline**							
*N*	410	118	160	96			
Age (years)	72.66 ± 6.95	75.21 ± 6.74	71.73 ± 7.20	73.14 ± 6.99	0.098 (62,667.0) (0.003)	< 0.001 (18,464.5) (0.029)	0.167 (6888.0) (0.007)
Gender (male %)	47.10	44.10	51.20	59.40	0.854 (0.034) (0.007)	0.564 (0.333) (0.025)	0.206 (1.596) (0.079)
Education (years)	16.65 ± 2.62	15.98 ± 2.65	15.99 ± 2.62	15.98 ± 2.61	0.016 (60,552.5) (0.007)	0.032 (21,101.0) (0.008)	0.723 (7479.5) (0.000)
*APOE* (ε4%)	42.20	56.80	37.50	60.40	0.866 (0.028) (0.002)	0.005 (7.861) (0.122)	< 0.001 (12.682) (0.223)
MMSE	28.69 ± 1.55	27.34 ± 1.93	28.48 ± 1.57	27.64 ± 1.75	0.030 (61,286.5) (0.006)	< 0.001 (14,213.5) (0.088)	< 0.001 (5480.0) (0.057)
ADAS-cog	12.54 ± 5.80	19.33 ± 7.40	12.23 ± 5.94	18.53 ± 7.53	0.315 (64,597.5) (0.001)	< 0.001 (11,618.5) (0.140)	< 0.001 (4001.5) (0.161)
FAQ	1.00 ± 2.38	3.55 ± 4.49	2.03 ± 3.47	4.88 ± 5.16	< 0.001 (48,915.0) (0.050)	< 0.001 (13,948.0) (0.093)	< 0.001 (4728.5) (0.103)
ADNI-MEM	0.75 ± 0.69	− 0.04 ± 0.67	0.62 ± 0.72	0.03 ± 0.68	0.001 (57,652.5) (0.014)	< 0.001 (10,177.5) (0.174)	< 0.001 (4280.0) (0.137)
MoCA	25.12 ± 2.92 (*n* = 335)	22.15 ± 2.77 (*n* = 59)	24.17 ± 3.10 (*n* = 126)	21.72 ± 2.93 (*n* = 58)	< 0.001 (27,283.5) (0.04)	< 0.001 (4464.5) (0.115)	< 0.001 (2095.0) (0.117)
Blood neutrophil (%)	60.48 ± 8.02 (*n* = 373)	62.00 ± 8.11 (*n* = 110)	60.47 ± 8.14 (*n* = 152)	64.06 ± 7.71 (*n* = 85)	0.850 (53,639.5) (0.003)	0.325 (18,143.0) (0.007)	0.010 (4929.0) (0.039)
CSF AD biomarker							
*N*	405	111	159	96			
Aβ_42_ (pg/ml)	1072.77 ± 452.07	705.17 ± 309.98	1044.35 ± 425.88	795.27 ± 395.27	1.070 (62,172.5) (0.002)	< 0.001 (11,012.5) (0.132)	< 0.001 (4617.5) (0.109)
t-tau (pg/ml)	238.35 ± 97.56	332.46 ± 115.14	231.89 ± 98.38	309.56 ± 123.64	0.915 (65,478.0) (0.000)	< 0.001 (11,534.5) (0.120)	< 0.001 (4596.0) (0.111)
p-tau (pg/ml)	22.32 ± 11.03	33.20 ± 12.84	21.71 ± 10.69	30.41 ± 14.13	1.018 (65,104.0) (0.000)	< 0.001 (10,913.5) (0.134)	< 0.001 (4594.5) (0.111)
CSF inflammation factor							
N	64	57	32	36			
TNFR2 (pg/ml)	1109.51.88 ± 636.53	1054.94 ± 308.02	948.15 ± 223.47	1091.54 ± 238.77	0.731 (3877.5) (0.002)	1.136 (1802.0) (0.000)	0.025 (347.0) (0.116)
TGFβ1 (pg/ml)	103.05 ± 30.40	104.31 ± 51.18	91.48 ± 27.43	119.11 ± 40.22	0.951 (4092.0) (0.000)	0.927 (1710.5) (0.003)	0.020 (327.5) (0.137)
ICAM1 (pg/ml)	369.10 ± 171.99	368.52 ± 166.55	310.57 ± 193.74	439.62 ± 253.14	0.488 (3646.5) (0.009)	1.019 (1804.0) (0.000)	0.020 (353.0) (0.110)
VCAM1 (ng/ml)	40.57 ± 17.52	47.25 ± 29.34	32.73 ± 11.53	42.30 ± 16.57	0.860 (3464.5) (0.017)	1.267 (1625.0) (0.009)	0.015 (353.0) (0.110)
TNFR1 (pg/ml)	879.43 ± 198.76	877.24 ± 253.18	800.46 ± 149.88	899.37 ± 210.30	0.797 (3858.0) (0.003)	1.006 (1695.0) (0.004)	0.095 (421.0) (0.053)
TGFβ2 (pg/ml)	156.04 ± 48.94	157.58 ± 50.13	149.73 ± 29.19	180.65 ± 57.13	0.620 (3637.0) (0.009)	0.944 (1810.5) (0.000)	0.052 (395.0) (0.073)
TGFβ3 (pg/ml)	9.06 ± 24.88	9.46 ± 24.74	6.27 ± 19.76	11.10 ± 27.89	0.824 (3818.0) (0.004)	0.375 (1482.0) (0.026)	0.439 (493.0) (0.015)
IL21 (pg/ml)	11.71 ± 11.50	11.86 ± 10.60	13.66 ± 15.37	10.69 ± 10.09	1.024 (4078.5) (0.000)	0.854 (1722.5) (0.002)	1.024 (568.0) (0.000)
IL7 (pg/ml)	1.13 ± 1.26	1.69 ± 3.22	1.18 ± 0.85	1.26 ± 1.13	1.115 (4065.0) (0.000)	0.550 (1455.0) (0.030)	0.922 (568.0) (0.000)
IL6 (pg/ml)	5.64 ± 6.55	5.17 ± 3.13	5.21 ± 6.22	4.78 ± 3.89	0.770 (3599.0) (0.011)	1.128 (1679.0) (0.005)	0.995 (555.0) (0.001)
**Annual rate of change**							
*N*	70	58	33	37			
Left hippocampus	1.02 ± 2.03	− 1.02 ± 2.52	1.60 ± 2.44	− 1.36 ± 3.15	1.402 (4332.0) (0.001)	< 0.001 (1065.0) (0.167)	< 0.001 (266.0) (0.235)
Right hippocampus	1.03 ± 3.14	− 0.68 ± 2.97	1.68 ± 4.30	− 1.73 ± 4.12	0.971 (4466.0) (0.000)	0.001 (1357.0) (0.081)	< 0.001 (291.0) (0.202)
**2-year follow-up**							
*N*	319	102	138	87			
MMSE	28.48 ± 1.92	24.91 ± 4.19	28.43 ± 1.81	25.62 ± 3.25	0.038 (42,882.0) (0.006)	< 0.001 (7112.5) (0.174)	< 0.001 (2664.0) (0.219)
ADAS-cog	11.95 ± 6.58	24.12 ± 11.44	11.82 ± 7.11	21.75 ± 10.28	0.306 (44,618.5) (0.002)	< 0.001 (5560.0) (0.238)	< 0.001 (2608.5) (0.226)
FAQ	1.02 ± 2.74	8.58 ± 8.16	2.50 ± 4.02	9.74 ± 8.66	< 0.001 (32,890.0) (0.057)	< 0.001 (5845.5) (0.226)	< 0.001 (2874.0) (0.192)
ADNI-MEM	0.84 ± 0.79 (*n* = 256)	− 0.36 ± 0.89 (*n* = 86)	0.78 ± 0.82 (*n* = 136)	− 0.23 ± 0.88 (*n* = 82)	0.052 (33,646.0) (0.007)	< 0.001 (3550.5) (0.258)	< 0.001 (2155.0) (0.264)
MoCA	25.40 ± 3.24 (*n* = 258)	20.00 ± 4.33 (*n* = 49)	24.78 ± 3.03 (*n* = 105)	20.78 ± 4.55 (*n* = 51)	0.002 (19,845.5) (0.02)	< 0.001 (1842.0) (0.201)	< 0.001 (1297.0) (0.174)

*MMSE* Mini-Mental State Examination, *ADAS-cog* Alzheimer’s Disease Assessment Scale-cognitive section, *FAQ* Functional Activities Questionnaire, *ADNI-MEM* ADNI memory, *MOCA* Montreal Cognitive Assessment, *CSF* cerebrospinal fluid, *Aβ* amyloid β, *p-tau* phosphor-tau, *t-tau* total tau, *TNFR* tumor necrosis factor receptor, *TGFβ* transforming growth factor-β, *ICAM1* intercellular cell adhesion molecule 1, *VCAM1* vascular cell adhesion molecule-1, *IL* interleukin

### Longitudinal effects of CSD on cognitive function and cognitive progression risk

A total of 784 individuals had in-person interviews at baseline and annual follow-up, with a total follow-up time of up to 168 months (*n* = 4253 person-times in total). Subjects with CSD displayed more longitudinal cognitive impairment and lower social activity function compared to normal sleep. There is no significant difference in the slope of the lines between the two groups (Fig. 1B1–B5).

During the follow-up period, in the CSD group, there were 35 stable normal cognitive subjects, 125 stable MCI subjects, 22 normal subjects developing MCI, and 74 MCI subjects developing dementia. In the normal sleep group, there were 224 stable normal cognitive subjects, 175 stable MCI subjects, 33 normal subjects developing MCI, 84 MCI subjects developing dementia, 1 normal subject developing dementia, and 11 MCI subjects reverting to normal. The results of the Kaplan–Meier analysis showed a significant difference in the cumulative proportion of individuals with cognitive stability between the normal sleep and CSD groups (*p* = 0.009) (Fig. 1C). The CSD group showed an increased risk of cognitive progression compared with the normal sleep group, according to the Cox proportional hazards model, with age, education years, gender, *APOE*, brain tau burden, baseline MMSE, and multiple psychiatric symptoms as covariates (Fig. 1D).

### Enrichment analysis in CSD elderly

**Fig. 2:**
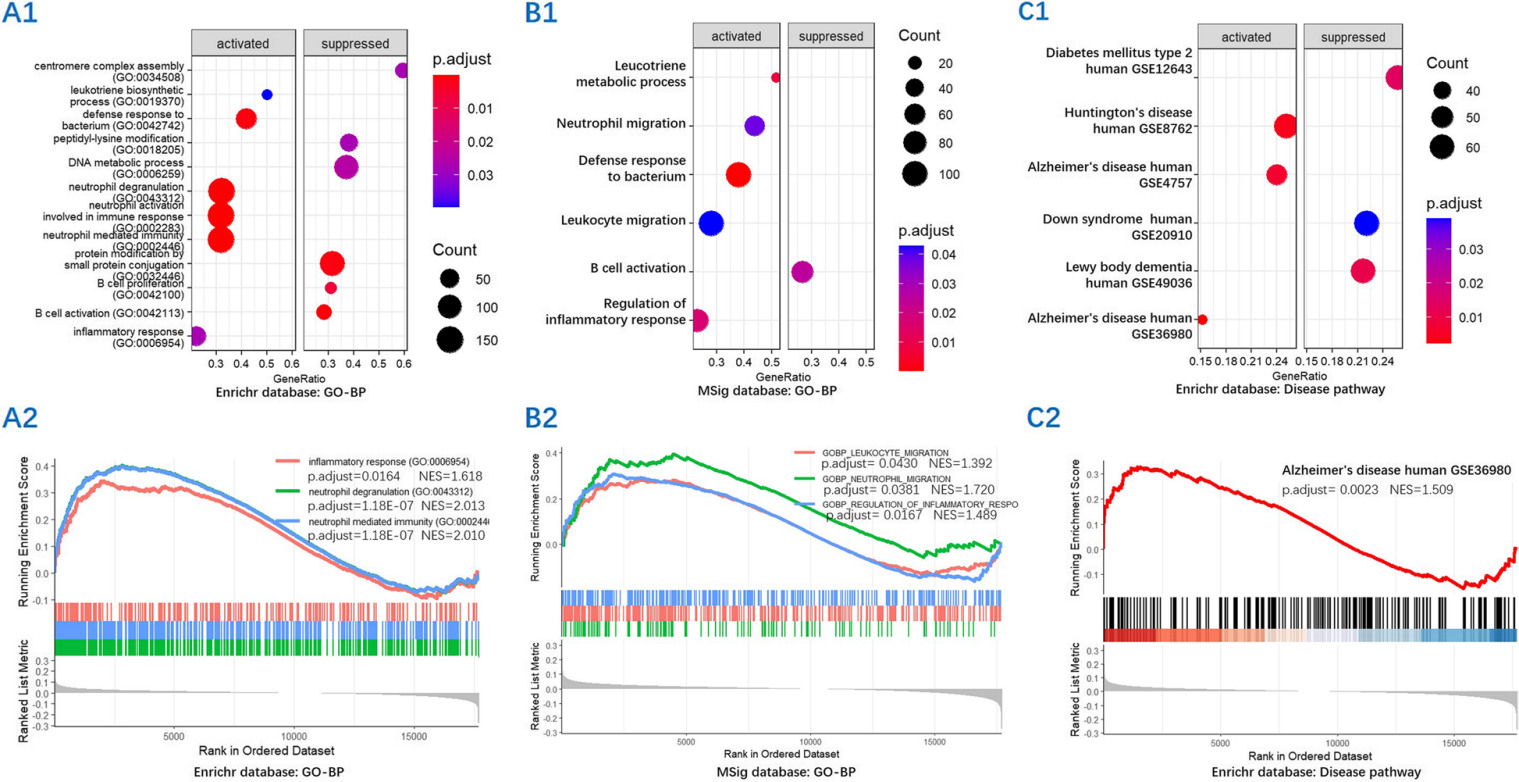
The activated neutrophil and AD pathology pathways for cognitively progressive in CSD through enrichment analysis. The GO-BP analysis showed the pathways related to neutrophil activation from the Enrichr database (**A1**, **A2**) and the MSig database (**C1**, **C2**), and the disease enrichment analysis showed the pathways related to AD pathology from the Enrichr database (**B1**, **B2**) in cognitively progressive subjects as compared to cognitively stable subjects with CSD

Among the CSD elderly, there were 88 cognitively stable and 54 cognitively progressive subjects. A total of 19,456 expressed genes were used for GSEA. GO biological process (BP) analysis showed that pathways related to neutrophil activation were significantly enriched in cognitively progressive subjects from the Enrichr (Fig. 2A1, A2) and MSig (Fig. 2C1, C2) databases. Disease pathway analysis showed that the AD-relevant pathway was significantly enriched in cognitively progressive subjects from the Enrichr database (Fig. 2B1, B2).

### Effects of blood neutrophil on cognitive progression risk

**Fig. 3:**
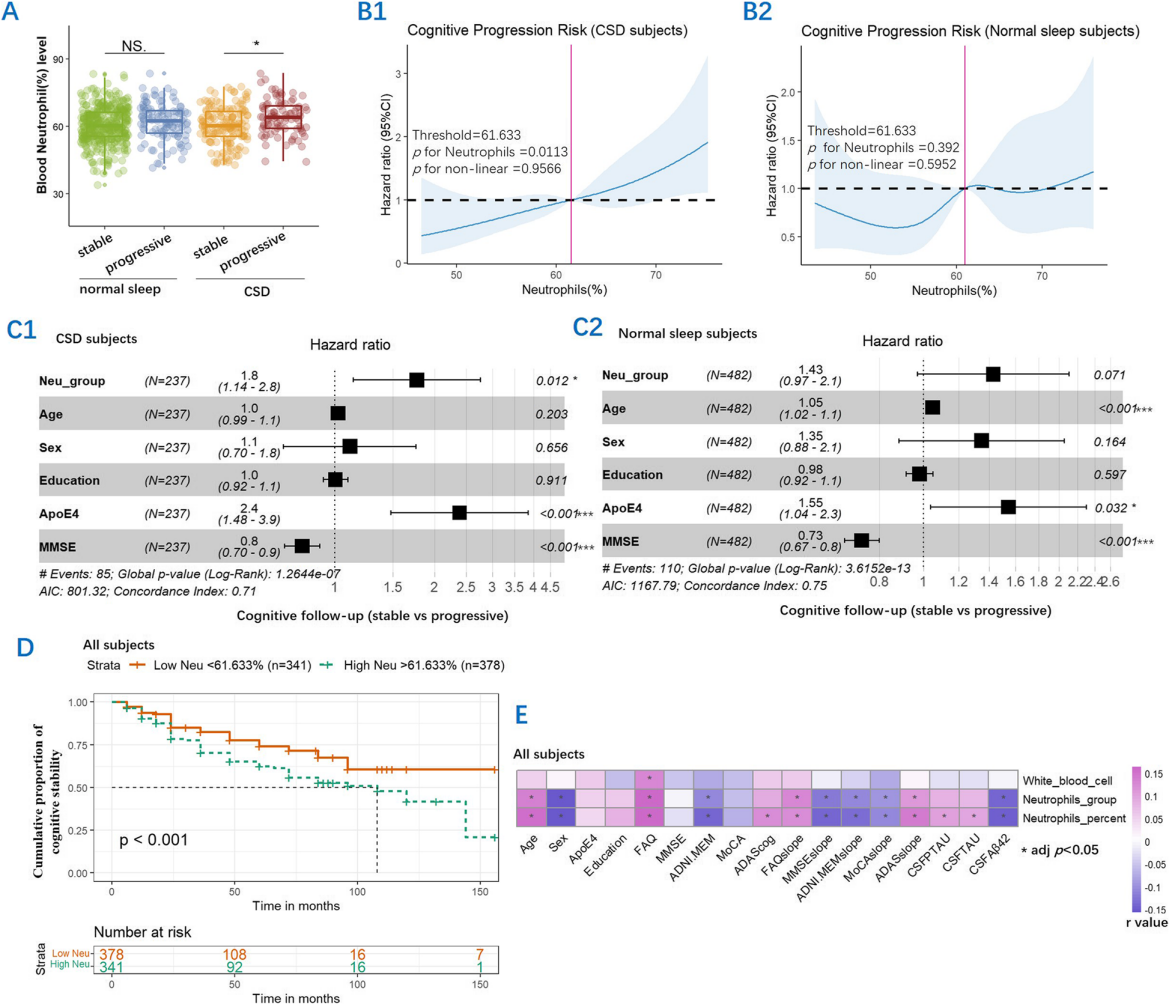
The significant differences in blood neutrophil percentage between the cognitively stable and cognitively progressive groups with CSD at baseline. NS adj *p* > 0.05, *adj *p* < 0.05 (**A**). The relative risk of cognitive progression was greater than 1.0 for the neutrophil (%) threshold of 61.633, and the relationship between neutrophil (%) and the corresponding HR was linear above this threshold in CSD subjects (**B1**), but there was no such relationship in normal sleep subjects (**B2**). Through the Cox proportional hazards model, blood neutrophil percentage was a risk factor for cognitive progression in the CSD group (**C1**) but not in the normal sleep group (**C2**). A Kaplan–Meier curve showing that subjects with higher blood neutrophil percentages were associated with a higher risk of cognitive progression (**D**). Spearman test showed that neutrophil percentage and neutrophil group correlated with CSF AD biomarker levels, baseline cognitive function, and annual changes in cognitive function, including ADAS-cog, MoCA, MMSE, FAQ, and MEM. *adj *p* < 0.05 (**E**)

A total of 720 participants had routine blood test of neutrophils, and 771 subjects had the CSF AD biomarker test. Cognitively progressive subjects with CSD had higher blood neutrophil percentage as compared with cognitively normal subjects with CSD; however, there was no difference in blood neutrophil percentage between cognitively progressive and cognitively normal subjects without CSD (Table 1) (Fig. 3A1) (adjusted *p* < 0.05). There were significant differences in Aβ, t-tau, or p-tau levels between cognitively normal and cognitively progressive subjects, in both normal sleep and CSD groups (Table 1) (adjusted *p* < 0.05) (Table 1). Hierarchical regression demonstrated the impact of neutrophil percentage on the annual change in cognitive function, including FAQ, MMSE, ADAS-cog, and ADNI-MEM in all the subjects (Table 2).

**Table 2:** Hierarchical regression in all the subjects

Dependent variable	Independent variables	Unstandardized coefficient (95% CI)	Standardized coefficient	Model	*R*^2^	*R*^2^ change	Sig *p* value
FAQ-slope	Age	0.002 (0.000, 0.003)	0.088				
	Education	− 0.005 (− 0.008, − 0.002)	− 0.106				
	Sex	− 0.013 (− 0.028, 0.003)	− 0.051				
	*APOE* ε4	0.057 (0.042, 0.072)	0.227	Model 1	0.078		
	Neutrophil (%)	0.002 (0.001, 0.003)	0.122	Model 2	0.092	0.014	< 0.001
ADAS-cog	Age	0.002 (0.001, 0.003)	0.097				
	Education	− 0.003 (− 0.006, 0.000)	− 0.056				
	Sex	− 0.008 (− 0.025, 0.009)	− 0.028				
	*APOE* ε4	0.074 (0.057, 0.090)	0.266	Model 1	0.085		
	Neutrophil (%)	0.001 (0.000, 0.002)	0.072	Model 2	0.090	0.005	0.023
MMSE-slope	Age	− 0.001 (− 0.002, 0.000)	− 0.103				
	Education	0.002 (0.001, 0.004)	0.091				
	Sex	0.006 (− 0.002, 0.015)	0.048				
	*APOE* ε4	− 0.030 (− 0.039, − 0.022)	− 0.227	Model 1	0.077		
	Neutrophil (%)	− 0.001 (− 0.001, 0.000)	0.106	Model 2	0.088	0.011	0.001
MEM-slope	Age	0.000 (0.000, 0.000)	− 0.103				
	Education	0.000 (0.000, 0.001)	0.091				
	Sex	0.001 (0.000, 0.002)	0.048				
	*APOE* ε4	− 0.005 (− 0.006, − 0.004)	− 0.227	Model 1	0.124		
	Neutrophil(%)	0.001 (0.000, 0.002)	− 0.106	Model 2	0.132	0.008	0.004

Model 1: demographics including age, sex, education, and *APOE*

Model 2: demographics and blood neutrophil percentage

RCS analysis was performed to further investigate the relationship between blood neutrophil and cognitive outcome, and the risk of cognitive progression increased with increasing blood neutrophil levels in CSD subjects (Fig. 3B1), with a threshold of 61.633 for blood neutrophil percentage. This such relationship was not observed in normal sleep subjects (Fig. 3B2). Individuals with higher neutrophil levels showed an increased risk of cognitive progression through the Cox regression, with age, education, sex, *APOE*, and baseline MMSE as covariates in CSD subjects (Fig. 3C1), but not in normal sleep subjects (Fig. 3C2). Survival analysis using the Kaplan–Meier curve revealed that subjects with higher neutrophil levels had a significant tendency for cognitive progression in all subjects (Fig. 3D). Significant correlations were also observed between blood neutrophil and annual changes in cognitive function, including MMSE, MEM, FAQ, MoCA, and ADAS-cog (adjusted *p* < 0.05) (Fig. 3E).

### Interaction and mediation analyses

**Fig. 4:**
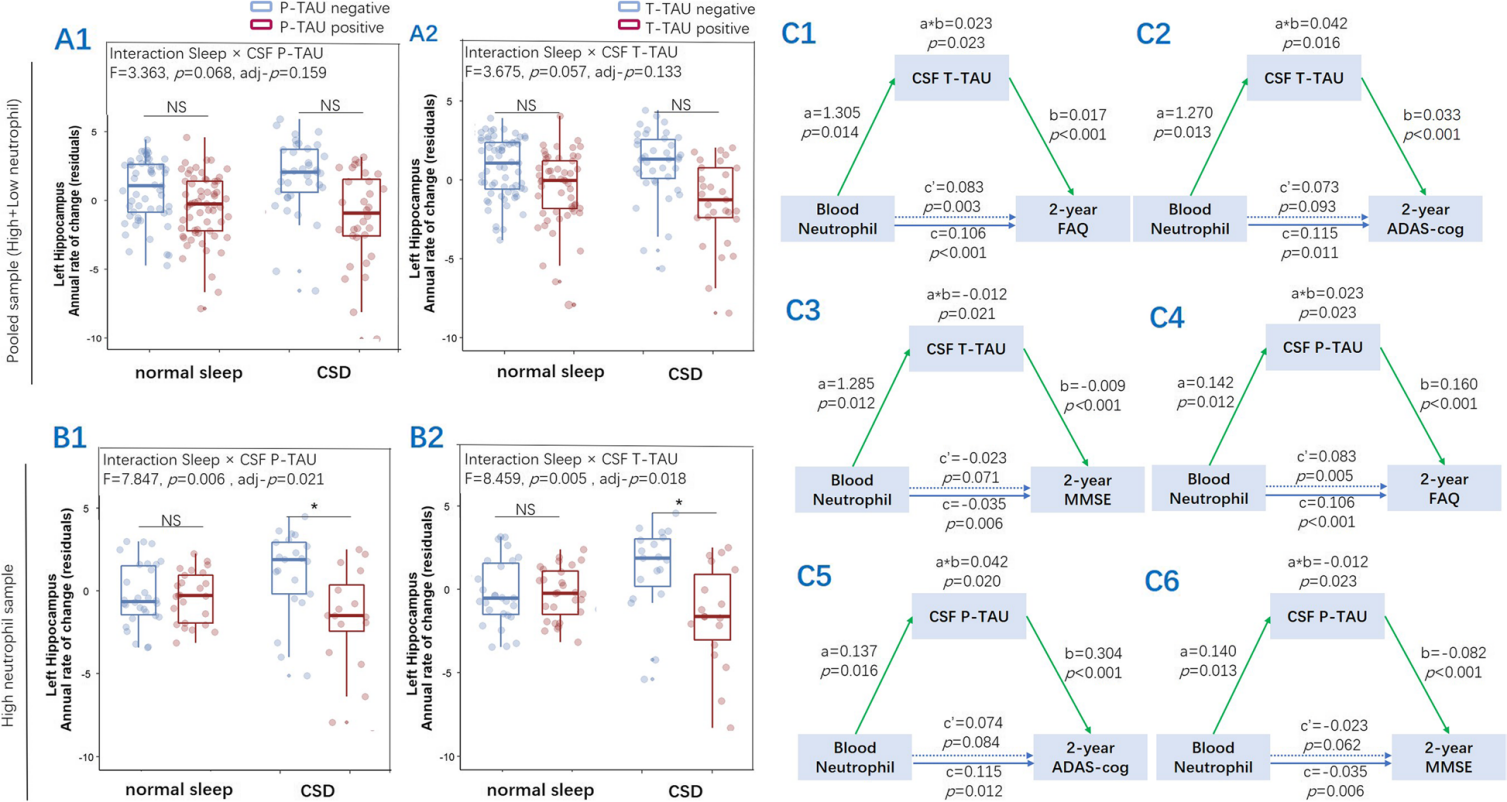
The interaction and mediation effects of blood neutrophil percentage on brain tau burden. The interaction effect of tau burden on longitudinal sleep-related changes in hippocampal volume was observed in the high neutrophil sample (**B1**, **B2**) but not in the pooled (high and low neutrophil) sample (**A1**, **A2**). NS adj *p* > 0.05, *adj *p* < 0.05. The mediation analysis showed the mediation effects of tau burden, including CSF t-tau (**C1**–**C3**) and p-tau (**C4**–**C6**), on blood neutrophil percentage within a 2-year follow-up of cognitive outcomes. The blue lines showed the total effect (*c*), the blue dotted lines showed the direct effect (*c*′), and the green lines depicted the mediation effects (a*b) of the tau burden. The path weight was expressed as an effect and a *p*-value

We investigated whether higher t-tau and p-tau levels exacerbated the effect of CSD on future hippocampal volume. Our findings showed a significant interaction between CSD and p-tau positivity (*F* = 7.847, adjusted *p* = 0.021) and interaction between CSD and t-tau positivity (*F* = 8.459, adjusted *p* = 0.018) on the annual change rate of left hippocampal volume in samples with higher neutrophil (Fig. 4B1, B2), but no significant results in all the samples (Fig. 4A1, A2), after controlling for the main effects of sleep, tau burden (t-tau or p-tau levels), and demographics. Furthermore, no such interaction was observed in the annual change rate of the right hippocampal volume. We found that t-tau and p-tau both mediated the association between blood neutrophil and cognitive function (ADAS-cog, MMSE, and FAQ) at 2 years follow-up. The mediation effect was considered to be partial, with the proportion of mediation ranging from 21 to 36% (Fig. 4C1–C6).

### Changes in inflammatory factors and correlation with tau burden due to CSD

**Fig. 5:**
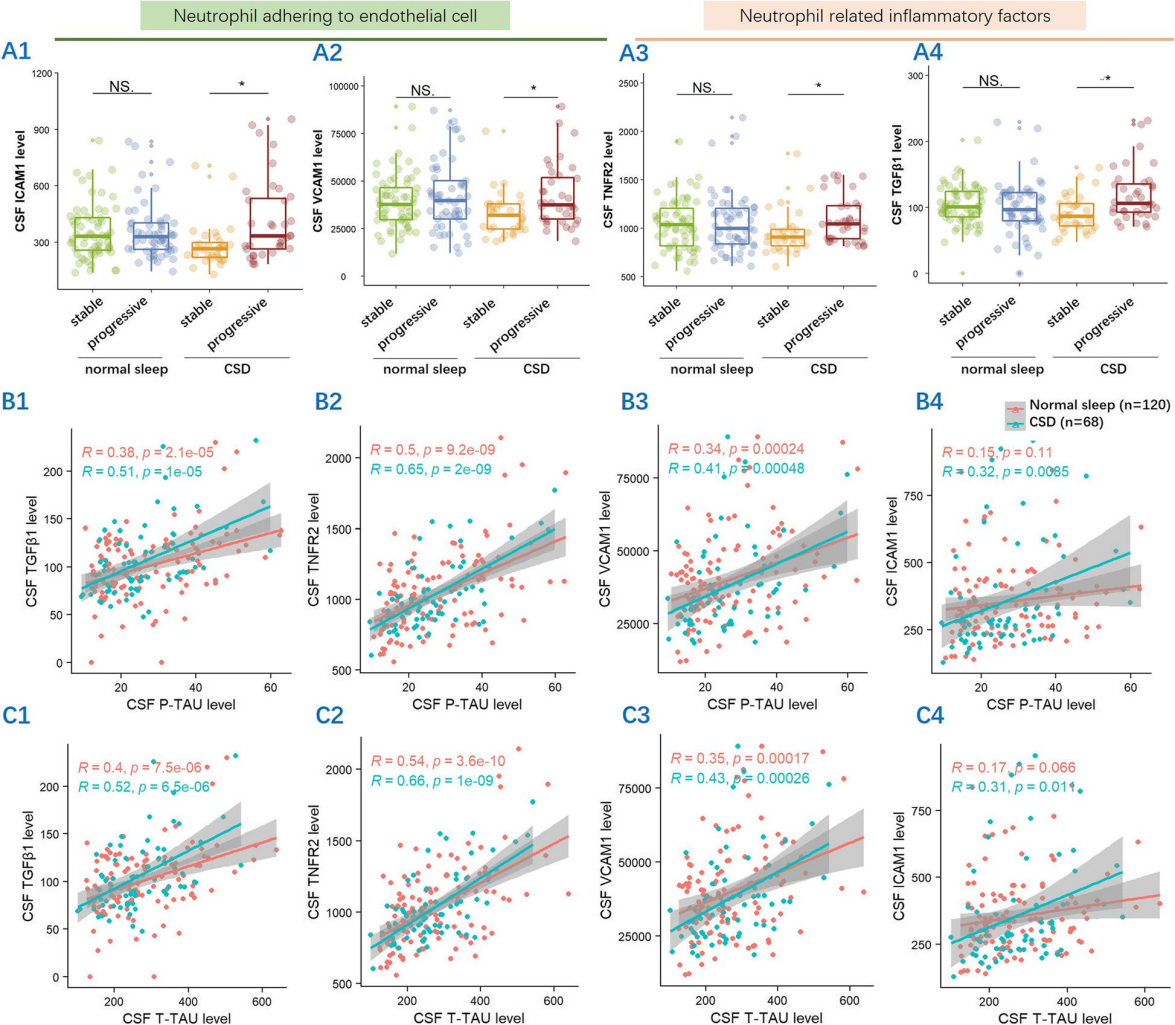
The difference in neutrophil-related inflammatory factors, including CSF ICAM1, VCAM1, TNFR2, and TGFβ1, between the cognitively stable and cognitively progressive groups in CSD elderly but not in normal sleep elderly. NS adj *p* > 0.05, *adj *p* < 0.01 (**A1**–**A4**). These four factors were significantly correlated with CSF p-tau levels (**B1**–**B4**) and CSF t-tau levels (**C1**–**C4**)

We assessed CSF inflammatory factors in 189 subjects and found that higher levels of inflammatory factors, including ICAM1, VCAM1, TNFR2, and TGFβ1, were present in cognitively progressive subjects with CSD (adjusted *p* < 0.05) but not in cognitively progressive subjects with normal sleep (Table 1, Fig. 5A1–A4). Other six inflammatory factors, including TNFR1; TGFβ2,3; IL-6,7,21, were not observed significantly different between the groups (Table 1). Correlation analysis showed that these four inflammatory factors were significantly associated with CSF p-tau (Fig. 5B1–B4) and t-tau (Fig. 5C1–C4) levels, especially in individuals with CSD (*p* < 0.05).

## Discussion

The present study had four main findings. First, CSD could significantly affect cognitive function. Second, we found significant effects of blood neutrophils on cognitive progression induced by CSD. Third, tau burden mediated the association of blood neutrophils with cognitive function and increased the CSD-related risk of left hippocampus atrophy. Fourth, neutrophil-related inflammatory factors were elevated and correlated with tau burden in cognitively progressive elderly with CSD.

Our results were consistent with previous longitudinal researches showing that CSD can predict cognitive progression [30, 31]. We found a significant difference in cognitive function between the normal sleep and CSD groups after 14 years of follow-up. Sleep difficulties have also been reported to link to an increased risk of AD. A study of 20 participants infused with C-leucine to measure Aβ kinetics showed that acute sleep deprivation increased the overnight Aβ38, Aβ40, and Aβ_42_ levels by 25 to 30% [32]. Pathologically high p-tau and gliosis were also detected in the hippocampus of CSD mice [33]. Unlike previous researches, we found no significant changes in Aβ, t-tau, or p-tau levels in CSD subjects, but only in cognitively progressive elderly, whether they had CSD or not. This finding suggests that AD pathology is not the main characteristic pathology of cognitive decline in CSD but is closely related to it.

The GSEA revealed neutrophil activation and AD pathology as potential pathways. Tauopathy, a typical pathological feature of AD, has been shown to cause sleep disturbance. Sleep disruption has also been observed in the transgenic mouse model with overexpressed human tau mutation [34]. Notably, we found significant changes in t-tau and p-tau levels in the cognitively progressive subjects with CSD. Relative to tau pathology, the activated neutrophil pathway was less studied. Previous studies showed that social stress upregulates inflammatory gene expression in the leukocyte transcriptome [35]. A study of partial night sleep deprivation also found a shift in leukocyte transcriptional profiles towards the expression of genes associated with cellular senescence [36]. We found that blood neutrophil was increased in cognitively progressive subjects with CSD. Similarly, the hyperactive neutrophil state has also been associated with the rate of cognitive progression [37]. According to the Framingham Heart Study, individuals with a higher neutrophil-to-lymphocyte ratio were at greater risk of subsequent dementia [38]. Our study found a significant risk of cognitive progression related to blood neutrophil levels in individuals with CSD.

Next, we investigated the interaction between the two pathways: AD pathology and neutrophil activation, and how this interaction contributed to the cognitive progression of CSD. The mediation analysis showed that t-tau and p-tau mediated the association between blood neutrophil and cognitive progression, and the effects varied from 21 to 36%. The interaction analysis showed that in the background of high blood neutrophil levels, high CSF t-tau and p-tau could exacerbate the effect of CSD on left hippocampal atrophy. Thus, we hypothesized that neutrophil-related neuro-immunity could possibly trigger or aggravate tau pathology, contributing to left hippocampal atrophy and subsequent cognitive progression in CSD.

Through hierarchical regression, we found that blood neutrophil impacted the annual change in cognitive function independently of CSF t-tau and p-tau levels, despite the small values of *R*^2^ change between the models with and without neutrophil. The possible mechanism is that neutrophil activation in CSD begins at the initial stage, then migrates to the perivascular space of the brain and triggers tau pathology at last, which maybe explain the small but significant effect of neutrophil on cognitive progression. Therefore, we predicted that neutrophil downstream inflammatory factors might have direct effects on cognitive progression through tau pathology. Neutrophils have a very short life cycle of up to 6 days in peripheral circulation [39]. In typical inflammation, neutrophils are cleared when the initial inflammatory insult is resolved. However, in CSD, neutrophils are recruited by chemokines, such as interleukin 8 (IL8) and C5a complement, and migrate into the peri-cerebrovascular space. As a result, neutrophils can adhere to endothelial cells, cross the blood–brain barrier, and release inflammatory factors that cause neuronal damage [40].

We found four factors that were significantly increased in CSF, including ICAM1, VCAM1, TNFR2, and TGFβ1. ICAM1 and VCAM1 required for neutrophil adhesion and trans-endothelial migration are highly expressed in endothelial cells [41]. TNFR2 is mainly expressed in immune cells, such as neutrophils, and acts as a potent pro-inflammatory cytokine. TGFβ1 has been implicated in myeloid cell activation, particularly neutrophil activation and degranulation [42]. Due to the neutrophil-adherent and inflammatory nature of these four factors, their upregulation suggests that the activation of the neutrophil downstream pathway occurs during the cognitive progression of CSD. Moreover, their levels correlated with CSF t-tau and p-tau, indicating that the activated neutrophil pathway was closely related to tau burden and brain function. Similar results from a large cohort study found that higher levels of CSF ICAM1 increased the risk of developing AD dementia and were associated with increased CSF levels of t-tau and p-tau [43]. Furthermore, chronic TNF treatment increased the release of toxic extracellular protein aggregates linking to AD pathology [44]. The above findings indicate that neutrophil-associated inflammatory cytokines may induce synergistic neurotoxicity that exacerbates neurodegeneration.

The present study had some limitations. Due to the lack of follow-up data on blood neutrophils, the trend of blood neutrophils in CSD was unclear. Also, only CSF t-tau and p-tau analysis was performed but not tau PET imaging, which limited the investigation of regional tau pathology in CSD. Additionally, this study included both cognitively normal and MCI subjects at baseline, which may have affected the population heterogeneity bias. So, the baseline cognitive function score has been incorporated into the Cox regression analysis to eliminate cognitive bias. Blood neutrophil and CSF tauopathy were identified as potential key pathways in the ADNI cohort, and future multi-cohort studies are needed for further validation.

## Conclusions

In summary, this study shows that CSD is associated with cognitive progression and that the activated neutrophil pathway is involved in this process. Neutrophil-related neuro-immunity may exacerbate tau pathology, contributing to cognitive progression in the context of CSD.

## Supplementary Information

**Additional file 1.** Supplementary description about included subjects and scale assessment.

**Additional file 2.** Flow chart of the inclusion and exclusion criteria.

**Additional file 3.** Supplementary methods for calculating hippocampal volume.

## Data Availability

ADNI is available at http://adni.loni.usc.edu.

## Abbreviations

AD: Alzheimer’s disease
ADAS-cog: Alzheimer’s Disease Assessment Scale-cognitive
ADNI: Alzheimer’s disease Neuroimaging Initiative
ANCOVA: Analysis of covariance
Aβ: β-Amyloid
BBB: Blood-brain barrier
BP: Biological process
CDR: Clinical Dementia Rating
CRP: C-reactive protein
CSD: Chronic sleep disturbance
CSF: Cerebrospinal fluid
EDTA: Ethylene diamine tetraacetic acid
FAQ: Functional Activities Questionnaire
GO: Gene Ontology
GSEA: Gene set enrichment analysis
HPA: Hypothalamus-pituitary-adrenal
ICAM1: Intercellular adhesion molecule 1
IL-1β: Interleukin 1β
IL6: Interleukin 6
IL-6: Interleukin 6
MCI: Mild cognitive impairment
MMSE: Mini-Mental State Examination
MoCA: Montreal Cognitive Assessment
NETs: Neutrophil extracellular traps
NPI: Neuropsychiatric Inventory
RCS: Restricted cubic spline
RFLP: Restriction fragment length polymorphism
RMA: Robust multichip average
SNS: Sympathetic nervous system
TGFβ 1: Transforming growth factor 1
TNFR1: Tumor necrosis factor receptor
TNF-α: Tumor necrosis factor α
VCAM-1: Vascular cell adhesion molecule-1
