# Supplementary material for: Neutrophil activation may trigger tau burden contributing to cognitive progression of chronic sleep disturbance in elderly individuals not living with dementia
Source: BMC Med. 2023 Jun 6;21:205. doi: 10.1186/s12916-023-02910-x (PMC10243051; doi:10.1186/s12916-023-02910-x)
Supplement: Supplementary file 1 — Additional file 1. Supplementary description about included subjects and scale assessment. [file 12916_2023_2910_MOESM1_ESM.docx]

**Subjects**

Neuropsychiatric symptoms were assessed with NPI, which is an informant-based instrument, that measures the presence (0=absent, 1=present), frequency, and severity (1=mild, 2=moderate, 3=severe) of several symptoms, including delusions, hallucinations, apathy, anxiety, sleep, depression, and so on. We used the presence or absence of sleep disturbance as our dichotomous indicator and divided all subjects into normal and CSD groups. The sleep disturbance questionnaire consisted of 8 questions, as follows:

1. Does the participant have difficulty falling asleep?

2. Does the participant get up during the night (do not count if the patient gets up once or twice per night only to go to the bathroom and falls back asleep immediately)?

3. Does the participant wander, pace, or get involved in inappropriate activities at night?

4. Does the participant awaken you during the night?

5. Does the participant awaken at night, dress, and plan to go out thinking that it is morning and time to start the day?

6. Does the participant awaken too early in the morning (earlier than was his/her habit)?

7. Does the participant sleep excessively during the day?

8. Does the participant have any other night-time behaviors that bother you that we haven't talked about?

In the ADNI, the selective criteria for cognitively normal, MCI and dementia subjects were as follows. Cognitively normal subjects had MMSE scores of 24-30, a Clinical Dementia Rating (CDR) of 0, normal memory function score on the Wechsler Memory Scale (WMS), a Memory Box (MB) score of 0, a Geriatric Depression Scale-15 (GDS-15) score of <7, no memory complaints, and an absence of significant impairment in cognitive functions or activities of daily living. MCI subjects had an MMSE score of 24-30, a CDR score of 0.5, abnormal memory function score on the WMS, an MB score of 0.5 or higher, a GDS-15 score of <7, an informant-reported memory complaint and objective evidence of memory loss, and general cognitive and functional performance such that a diagnosis of AD could not be made by the site physician. Through follow-up, dementia was diagnosed with an MMSE score of 20-26, a CDR score of 0.5 or 1.0, abnormal memory function score on the WMS, a GDS-15 score of <7, and subjective memory complaints reported by the subject, study partner, or clinician.
