# Supplementary figures and images for: Neutrophil activation may trigger tau burden contributing to cognitive progression of chronic sleep disturbance in elderly individuals not living with dementia
Source: BMC Med. 2023 Jun 6;21:205. doi: 10.1186/s12916-023-02910-x (PMC10243051; doi:10.1186/s12916-023-02910-x)

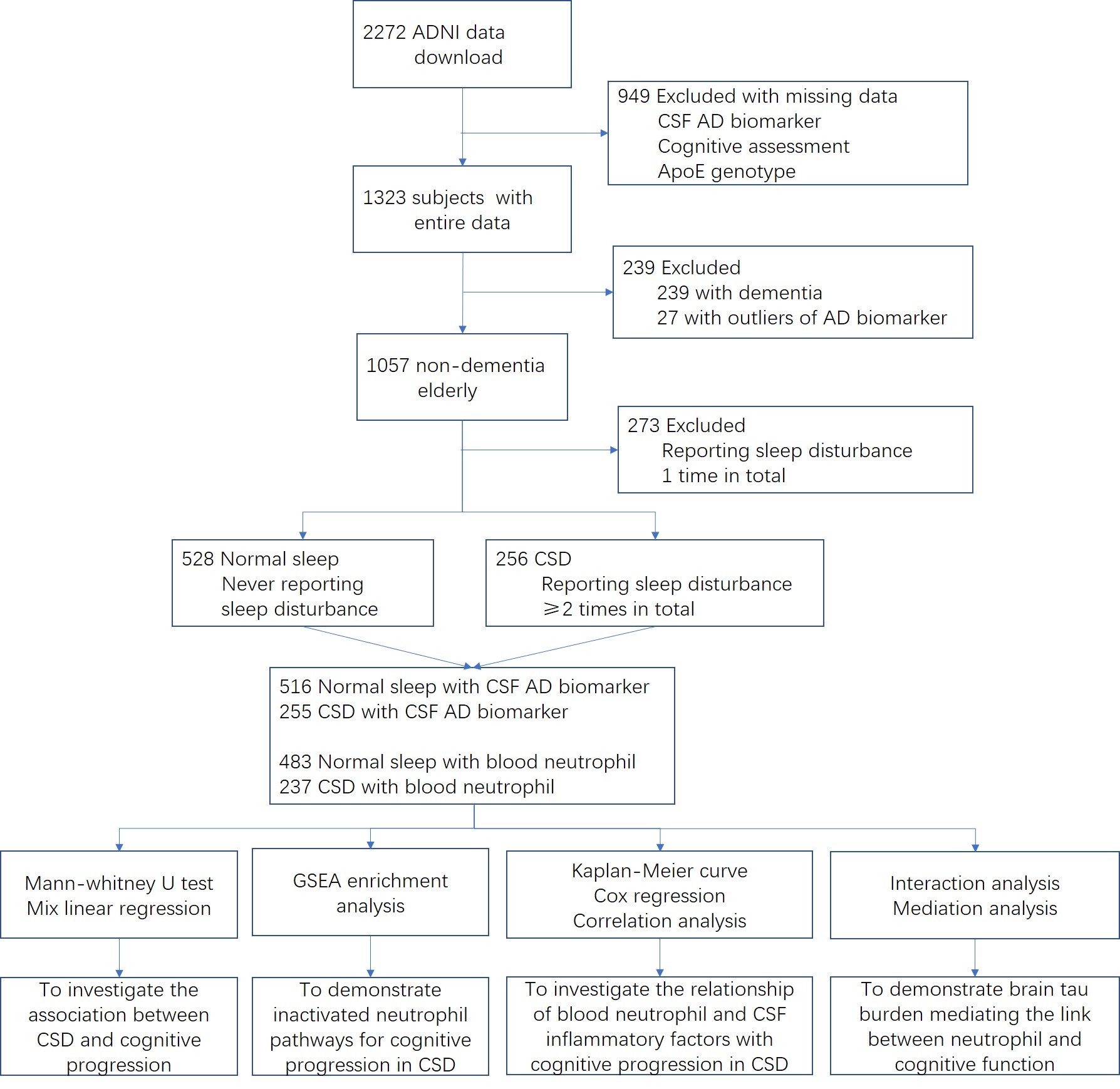

Supplement: Supplementary file 2 — Additional file 2. Flow chart of the inclusion and exclusion criteria. [file 12916_2023_2910_MOESM2_ESM.jpg]
