# Supplementary material for: Neutrophil activation may trigger tau burden contributing to cognitive progression of chronic sleep disturbance in elderly individuals not living with dementia
Source: BMC Med. 2023 Jun 6;21:205. doi: 10.1186/s12916-023-02910-x (PMC10243051; doi:10.1186/s12916-023-02910-x)
Supplement: Supplementary file 3 — Additional file 3. Supplementary methods for calculating hippocampal volume. [file 12916_2023_2910_MOESM3_ESM.docx]

**Hippocampal volume**

Annual rates of change in hippocampal volumes were measured longitudinally in 198 subjects with ≥3 available MRI scans provided by ADNI imaging data. Hippocampal volumes measured using 3T structural MRI and the protocols from Fox Lab - BSI Measures Methods, are available online (<http://adni.loni.usc.edu/>).

Boundary shift integral (BSI) was used as the processing method. The brain and ventricles were automatically delineated from the T1-weighted MRI scans. The repeat scans were then registered to the baseline scans using 9-degree-of-freedom registration. Intensity inhomogeneity between the baseline and registered repeat scans was corrected using the differential bias correction. Finally, the BSI was calculated over the boundaries of the brain and ventricle respectively using the registered and corrected scans. The indices were obtained by the method including whole brain volume, ventricular volume, and hippocampal volume. The corrected right and left hippocampal volumes were calculated from the ratio of hippocampal volume to total brain volume. To determine annual rates of change in hippocampal volume, we used the fitted linear mixed models with hippocampal volume as the dependent variable and time (years from baseline) as the independent variable, controlling for random intercept and slope. Then, a slope for the annual rate of change was generated for each subject.
